# *Wohlfahrtiimonas chitiniclastica* Monomicrobial Bacteremia in a Homeless Man

**DOI:** 10.3201/eid2712.210327

**Published:** 2021-12

**Authors:** Omar Harfouch, Paul M. Luethy, Mandee Noval, Jonathan D. Baghdadi

**Affiliations:** University of Maryland Medical Center, Baltimore, Maryland, USA

**Keywords:** *Wohlfahrtiimonas chitiniclastica*, sepsis, skin, soft tissue, infection, bacterial infection, bacteria, bacteremia, bloodstream infections, Maryland, United States

## Abstract

We report a case of septic shock attributable to monomicrobial bloodstream infection secondary to *Wohlfahrtiimonas chitiniclastica* infection. This case suggests that *W. chitiniclastica* likely possesses the virulence to cause severe disease. Culture-independent techniques were essential in the identification of this organism, which enabled selection of appropriate therapy.

In August 2020, a 63-year-old homeless man with a history of deep vein thrombosis and chronic venous insufficiency was found in his truck, unconscious and covered in feces and maggots. He reportedly had been parked in a single parking spot in rural Maryland, USA, for 3 days. His blood pressure in the field was too low to be quantified, and he was admitted to a community hospital in septic shock. Blood cultures were drawn before establishing intravenous access for administration of vancomycin, piperacillin/tazobactam, and crystalloid. After being stabilized, he was transferred to our hospital, a tertiary care center in Baltimore, Maryland, USA, where surgeons performed superficial surgical debridement of his lower extremities and removed maggots by using a scrub brush with the patient under anesthesia in the operating room. We discarded the maggots, and they were not submitted for identification. 

The patient’s leukocyte count on arrival was 38.6 K/μL (reference range 4.5–11.0 K/μL), his creatinine 6.86 mg/dL (reference range 0.7–1.5 mg/dL), and his lactic acid 3.5 mmol/L (reference range 0.5–2.2 mmol/L). He had elevated transaminases, an aspartate aminotransferase level of 436 U/L (reference range 17–59 U/L) and alanine transaminase of 174 U/L (reference range 0–49 U/L). A computed tomography scan of the lower extremities showed ulceration of the anterior right lower leg with edema and fat stranding of the subcutaneous tissue without fluid collection or gas. A magnetic resonance imaging of his left foot showed no evidence of osteomyelitis.

On day 2 of hospitalization, transient hemodynamic instability necessitated initiation of vasopressor support and continuous renal replacement therapy; however, these treatments were rapidly tapered off. We identified gram-negative rods in the anaerobic blood culture from the community hospital, and we narrowed the patient’s antibiotics to piperacillin/tazobactam monotherapy. On hospital day 5, we identified the gram-negative rods as *W. chitiniclastica* by using matrix-assisted laser desorption/ionization time-of-flight (MALDI-TOF) mass spectrometry. We changed the patient’s intravenous antibiotics to 2 g of ceftriaxone daily and then, on hospital day 9, changed the regimen to 750 mg of oral levofloxacin daily to complete a 21-day course of treatment. We were unable to follow up with the patient after his discharge, but we proceeded with reporting about his case after it was deemed to be exempt by the Institutional Review Board at the University of Maryland Baltimore.

In 2008, *W. chitiniclastica* was first isolated from larvae of the parasitic fly *Wohlfahrtia magnifica* (*1*). Since 2008, a total of 11 cases of *W. chitiniclastica* bloodstream infections have been described (*2*–*10*; Appendix references *11,12*) (Table). Our patient shares risk factors observed in other cases, including homelessness and chronic venous insufficiency (Appendix reference *13*). The pathogenicity of *W. chitiniclastica* has remained uncertain in previous case reports secondary to its identification in polymicrobial infections. This severe case of monomicrobial *W. chitiniclastica* BSI is similar to a previous report of a 70-year-old man in Argentina who had septic shock with multiorgan failure secondary to the same bacteria (*3*). Taken together, these 2 cases challenge the hypothesis that other bacteria present in polymicrobial infections are primarily responsible for the disease associated with BSI attributable to *W. chitiniclastica* infection (*9*) and instead suggest that this pathogen may cause severe disease.

**Table:** Published cases of *Wohlfahrtiimonas* chitiniclastica bloodstream infection*

Country of origin (reference)	Age, y/sex; housing status; presentation	Bacteria identified on blood cultures	Microbiology tools used	Antimicrobial agents and duration of treatment	Outcome
France (*2*)	60/F; homeless; fatigue and ulcers to the scalp	*W. chitiniclastica*	16S rRNA sequencing	Ceftriaxone; duration not defined	Survival
Argentina (*3*)	70/M; homeless; altered mental status, septic shock, and plaques in the inguinal region	*W. chitiniclastica*	16S rRNA sequencing	Ciprofloxacin and ampicillin/sulbactam; duration not defined	Death
Washington, USA (*4*)	57/M; stable home; wet gangrene of the ankle, septic shock, and multi-organ failure	Propionibacterium acnes, *Staphylococcus hominis*, and *Wohlfahrtiimonas* species	MALDI-TOF mass spectrometry and 16S rRNA sequencing	No mention of antimicrobials used	Death
Ohio, USA (*5*)	41/F; stable home; abdominal pain and sacral osteomyelitis	*Proteus mirabilis* and *W. chitiniclastica*	MALDI-TOF mass spectrometry	Vancomycin, cefepime, and metronidazole; duration of 6 wks	Death from *Clostridioides difficile* infection
Indiana, USA (*6*)	37/M; not specified; necrotizing infection of lower extremities	*W. chitiniclastica*, *Ignatzschineria* *Indica*, and *Providencia stuartii*	Not specified	Piperacillin/tazobactam, clindamycin and vancomycin, then cefepime; duration of 10 d	Survival
United Kingdom (*7*)	82/F; stable home; unconscious	*W. chitiniclastica*, *P. mirabilis*, *Providencia rettgeri*, and *Staphylococcus aureus*	MALDI-TOF mass spectrometry and 16S rRNA sequencing	Cefuroxime, metronidazole, and clarithromycin, then flucloxacillin; duration of 7 d	Survival
Australia (*8*)	54/M; stable home; unconscious, septic shock and myasis of the foot and toes	*W. chitiniclastica* and *Morganella morganii*	MALDI-TOF mass spectrometry	Piperacillin/tazobactam, then meropenem, then ciprofloxacin; duration of 3 wks	Survival
Hawaii, USA (*9*)	72/M; stable home; unconscious, septic shock, and myasis of the umbilical cord	*Escherichia coli* and *W. chitiniclastica*	16S rRNA sequencing	Piperacillin/tazobactam, clindamycin, and vancomycin; duration not specified	Death
Japan (*10*)	75/M; homeless; unconscious	*Peptoniphilus harei* on initial blood cultures. On day 20, *P. mirabilis*, *M. morganii*, *Streptococcus anginosus*, *Streptococcus agalactiae*, *Bacteroides fragilis*, and *W. chitiniclastica*	MALDI-TOF mass spectrometry and 16S rRNA sequencing	Cefazolin, then vancomycin, cefepime, and metronidazole; duration not specified	Survival
North Dakota, USA (Appendix reference *11*)	70/M; stable home; fall	*W. chitiniclastica*	Not specified	Levofloxacin; duration not specified	Survival
Pennsylvania, USA (Appendix reference *12*)	82/M; stable home; fall and confusion, myasis of the lower extremities and toes	*Staphylococcus aureus*, *W. chitiniclastica*, and *I. indica*	MALDI-TOF mass spectrometry	Daptomycin for 6 wks Ceftriaxone for 2 wks	Survival

*Appendix. MALDI-TOF, matrix-assisted laser desorption/ionization time-of-flight.

For our patient, *W. chitiniclastica* was first identified on MALDI-TOF mass spectrometry from a positive anaerobic blood culture. In all 9 cases for which detailed microbiologic methods are reported, *W. chitiniclastica* was identified from blood or tissue cultures by using MALDI-TOF mass spectrometry (*5*,*8*; Appendix reference *12*), 16S rRNA sequencing (*2*,*3*,*9*), or both (*4*,*7*,*10*) (Table). This pattern demonstrates that *W. chitiniclastica* is extremely difficult to identify from clinical specimens without culture-independent techniques and highlights the utility of these techniques in clinical care.

Published case-reports demonstrate a heterogeneous approach to the clinical management of patients with *W. chitiniclastica* BSI. Often, selection of antibiotics was dictated by the other pathogens present in a polymicrobial infection. Generally, most studies report the use of β-lactams (*2*,*3*,*5*–*10*; Appendix reference *12*) as initial therapy, with fluoroquinolones available as second-line or step-down therapy (*3*,*7*,*8*). The duration of treatment ranges from 7 days to 6 weeks (*5*–*8*; Appendix reference *12*). Given that our patient rapidly improved and the presumed source of his infection had been controlled with debridement of his lower extremities, we opted for a 3-week course of treatment.

Appendix Additional information about *Wohlfahrtiimonas chitiniclastica* monomicrobial bacteremia in a homeless man.
