# Supplementary material for: Wohlfahrtiimonas chitiniclastica Monomicrobial Bacteremia in a Homeless Man
Source: Emerg Infect Dis. 2021 Dec;27(12):3195–7. doi: 10.3201/eid2712.210327 (PMC8632184; doi:10.3201/eid2712.210327)
Supplement: Appendix — Additional information about Wohlfahrtiimonas chitiniclastica monomicrobial bacteremia in a homeless man. [file 21-0327-Techapp-s1.pdf]

Article DOI: https://doi.org/10.3201/eid2712.210327

Wohlfahrtiimonas chitiniclastica Monomicrobial Bacteremia in a Homeless Man

Appendix

References

11. Bueide P, Hunt J, Bande D, Guerrero DM. Maggot wound therapy associated with *Wohlfahrtiimonas chitiniclastica* blood infection. Cureus. 2021;13:e12471. PubMed

12. Snyder S, Singh P, Goldman J. Emerging pathogens: A case of *Wohlfahrtiimonas chitiniclastica* and *Ignatzschineria indica* bacteremia. IDCases. 2020;19:e00723. PubMed https://doi.org/10.1016/j.idcr.2020.e00723

13. Schröttner P, Rudolph WW, Damme U, Lotz C, Jacobs E, Gunzer F. *Wohlfahrtiimonas chitiniclastica*: current insights into an emerging human pathogen. Epidemiol Infect. 2017;145:1292–303. PubMed https://doi.org/10.1017/S0950268816003411
